# Meta-analysis of the efficacy of Er Chen Tang in the adjuvant treatment of obesity: A study protocol for systematic review and meta-analysis

**DOI:** 10.1097/MD.0000000000038504

**Published:** 2024-06-07

**Authors:** Xueyan Lv, Xingyu Kang

**Affiliations:** a The First Clinical Medical College of Heilongjiang University of Chinese Medicine, Haerbin, Heilongjiang Province, China; b The Second Clinical Medical College of Heilongjiang University of Chinese Medicine, Haerbin, Heilongjiang Province, China.

**Keywords:** Er Chen Tang, meta-analysis, obesity, prospective study

## Abstract

**Objective:**

To systematically evaluate the efficacy of Er Chen Tang in the adjuvant treatment of obesity.

**Methods:**

A computerized search of databases such as CNKI, Wanfang, Wipro, EMBase, Web of Science, PubMed, and Cochrane Library was performed to collect randomized controlled trials on the application of Er Cheng Tang for the treatment of obesity and to track the references included in the literature, with a timeframe from the establishment of the library to October 2023 for the searches. After selection of trials, extraction of information and assessment of methodological quality were done independently by 2 evaluators, meta-analysis was performed using RevMan 5.3 software and the quality of evidence was evaluated using the Cochrane system.

**Results:**

Six studies were included, with a total of 438 study participants. They were randomized into trial and control groups. The total cholesterol, triglyceride, low-density lipoprotein cholesterol, body mass index, and visceral fat area values before treatment were compared between the 2 groups, and the differences were not statistically significant (all *P* > .05). After treatment, the indicators of the experimental group were significantly better than those of the control group, and the differences were all statistically significant (*P* < .05).

**Conclusion:**

The adjuvant treatment of obesity with Er Chen Tang can improve the symptoms faster and is favorable to the reduction of various risk indicators. However, due to the lack of high-quality literature, the theoretical support of large-sample double-blind randomized trials is still needed in the future.

## 1. Introduction

Obesity is a weight gain of more than 20% above ideal body weight caused by excessive fat storage or abnormal distribution in the body. Obesity is a chronic metabolic disease caused by the interaction of genetic and environmental factors, which can lead to a variety of metabolism-related diseases. The World Health Organization now characterizes obesity as the world’s largest chronic disease. Weight loss is not only to make the body beautiful, but also to promote health and reduce additional medical expenses.^[1]^ Er Chen Tang is a widely known traditional Chinese medicine formula from “Taiping Huimin Hejiaobu Fang,” which has the effects of strengthening the spleen, inducing diuresis, resolving phlegm, and regulating qi. As a classic phlegm-reducing soup, Er Chen Tang has a good regulatory effect on fatty acid metabolism.^[2]^ Therefore, this study collected data from clinical trials on the adjuvant treatment of obesity with Er Chen Tang, and objectively evaluated the therapeutic efficacy of using Er Chen Tang for the adjuvant treatment of obesity with the use of meta-analysis, with the expectation of providing reliable medical evidence. In the past, there have been some clinical trials on this study in China, but the number is small, especially the lack of high-quality literature, and many researchers do not fully recognize the utility of this, we look forward to this meta-analysis to broaden the ideas for future research and contribute to the promotion of human health. It is worth noting that the studies included in the paper were supplemented with Er Chen Tang as an adjuvant to conventional therapies, not as a replacement for the original therapy.

## 2. Information and methodology

### 2.1. Sources

#### 2.1.1. Literature inclusion criteria

Randomized controlled trials (RCT) on the adjuvant treatment of obesity with Er Chen Tang, with complete literature; literature reported the diagnosis and basis of obesity; comparability between the test group and the control group.

#### 2.1.2. Exclusion criteria

The source of the research subjects mentioned in the literature is not clear; the result data in the literature is incomplete and the reason is not explained; the same literature in different databases.

### 2.2. Methodology

#### 2.2.1. Search strategy

Erchen, Decoction, Obesity, Fat, RCT were used as the English search terms, and Erchen Tang, Obesity, Hyperlipidemia, RCT were used as the Chinese search terms, and the combination of subject terms and free terms was used for the search. Computerized searches of major academic literature databases at home and abroad, such as CNKI, Wanfang, Wipro, PubMed, EMBase, Cochrane Library, and Web of Science, were used to find RCTs on the application of Er Chen Tang for the adjuvant treatment of obesity and to track the references of the included RCTs, and the timeframe for the literature searches was from the establishment of the database to October 2023 for all of them.

#### 2.2.2. Screening the literature and extracting data

The initial screening was carried out by 2 researchers: each of them independently read the titles and abstracts of the literature, excluded duplicates, excluded literature that did not meet the inclusion criteria, and read the full text of the literature that met the inclusion criteria. The 2 researchers checked the results of the included literature with each other, carried out quality assessment, and if there was any disagreement, a 3rd researcher was consulted to reach a consensus. Data were extracted on: time of publication; inclusion and exclusion criteria; general information about the members of the trial and control groups; interventions; and test indicators. If the included RCTs had incomplete late outcome data, the reason for data loss should be found in the original article.

#### 2.2.3. Literature quality evaluation

Two researchers will evaluate the quality of RCTs that are screened and decided to be included in the analysis by referring to the “Cochrane Collaboration’s Risk of Bias Assessment Criteria.” Specific content should include: random allocation method, masking of allocation scheme, blinding, blinding evaluation of outcomes, completeness of outcome data, and selective reporting of studies.

#### 2.2.4. Conclusion

Indicators: total cholesterol (TC); triglycerides (TG); low-density lipoprotein cholesterol (LDL-C); body mass index (BMI); visceral fat area (VFA).

### 2.3. Statistical analysis

Meta-analysis was performed using RevMan 5.3 software. Because the units were the same, the measurement data were evaluated by mean difference (MD), and the count data were evaluated by relative risk (RR), and the heterogeneity was determined by the *I*^2^ test, *I*^2^ < 25% for no heterogeneity, 25% < *I*^2^ < 50% for mild heterogeneity, all of which indicated that the differences were not statistically significant, and the meta-analysis applied the fixed effect model; 50% < *I*^2^ < 75% is moderate heterogeneity, *I*^2^ > 75% is severe heterogeneity, all indicate statistically significant differences, meta-analysis applies random-effects model, while sensitivity analysis, subgroup analysis should be performed to explain the source of heterogeneity. Meta-analysis applies funnel plot to identify publication bias.

## 3. Results

### 3.1. Literature search results

**Figure 1.**
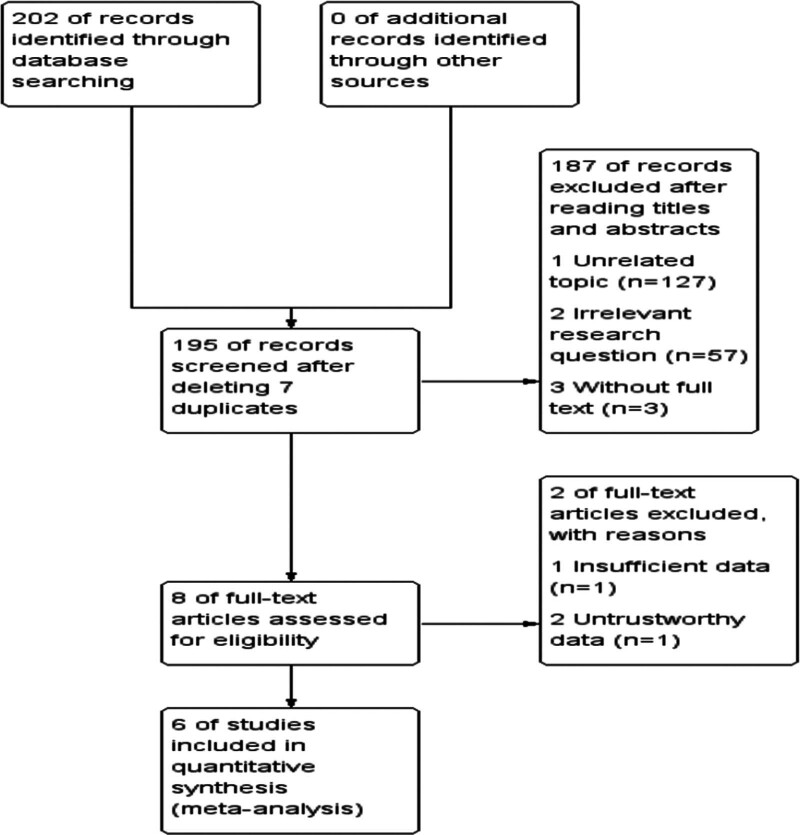
Literature screening process.

A total of 202 papers were initially examined, duplicates and papers that did not meet the inclusion requirements were excluded, and a total of 6 papers were finally included after reading the title, abstract and body in order. There were a total of 438 patients, including 218 cases in the control group and 220 cases in the experimental group. The process of literature screening is shown in Figure 1, and the basic information of inclusion analysis is shown in Table 1.

**Table 1:** Basic characteristics of the 6 studies included in the efficacy meta-analysis.

First author	Year of publication	Inclusion number of cases		Age (yrs)		Intervention		Outcome
		Test group	Control group	Test group	Control group	Test group	Control group	
Yao ^[3]^	2019	30	30	7 to 14	7 to 14	Er Chen Tang, 1 dose/d, orally + external treatment at auricular points + Dietary control + physical activity, course of 90 d	Dietary control + physical activity, course of 90 d	④
Zeng ^[4]^	2007	32	30	34 to 78 (58.6 ± 5.7)	36 to 75 (54.6 ± 6.5)	Hematopoeia and Blood Stasis Tang combined with Er Chen Tang, 1 dose/d, orally + Metformin tablets, 1.5 g/d, orally for 60 d	Metformin tablets, 1.5 g/d, orally for 60 d	①②④
Liu^[5]^	2023	38	38	36 to 69 (53.65 ± 3.42)	38 to 68 (54.16 ± 3.53)	Er-ju Er Chen Tang, 250 mL/d, orally + Baduanjin + Dagliflozin, 10 mg/d orally for 90 d	Dagliflozin, 10 mg/d orally for 90 d	④⑤
Zheng^[6]^	2022	32	32	49.53 ± 8.76	50.75 ± 7.03	Er-ju Er Chen Tang, 250 mL/d, orally + Baduanjin + Dagliflozin, 10 mg/d orally for 90 d	Dagliflozin, 10 mg/d orally for 90 d	④⑤
Chen^[7]^	2022	50	50	8 to 15 (11.28 ± 3.31)	7 to 15 (10.35 ± 3.22)	Er Chen Tang, 2 times/d+口服+Aerobic exercise, 5 to 7 times/week for 60 d	Aerobic exercise, 5 to 7 times/week for 60 d	①②③④
Lu^[8]^	2021	38	38	45.60 ± 11.33	45.60 ± 11.33	Free and easy san combined with Er Chen Tang, 1 dose/d, orally + HAART treatment for 90 d	HAART treatment for 90 d	①②③

Tip: ① TC; ② TG; ③ LDL-C; ④ BMI; ⑤VFA.

### 3.2. Literature quality evaluation

**Figure 2.**
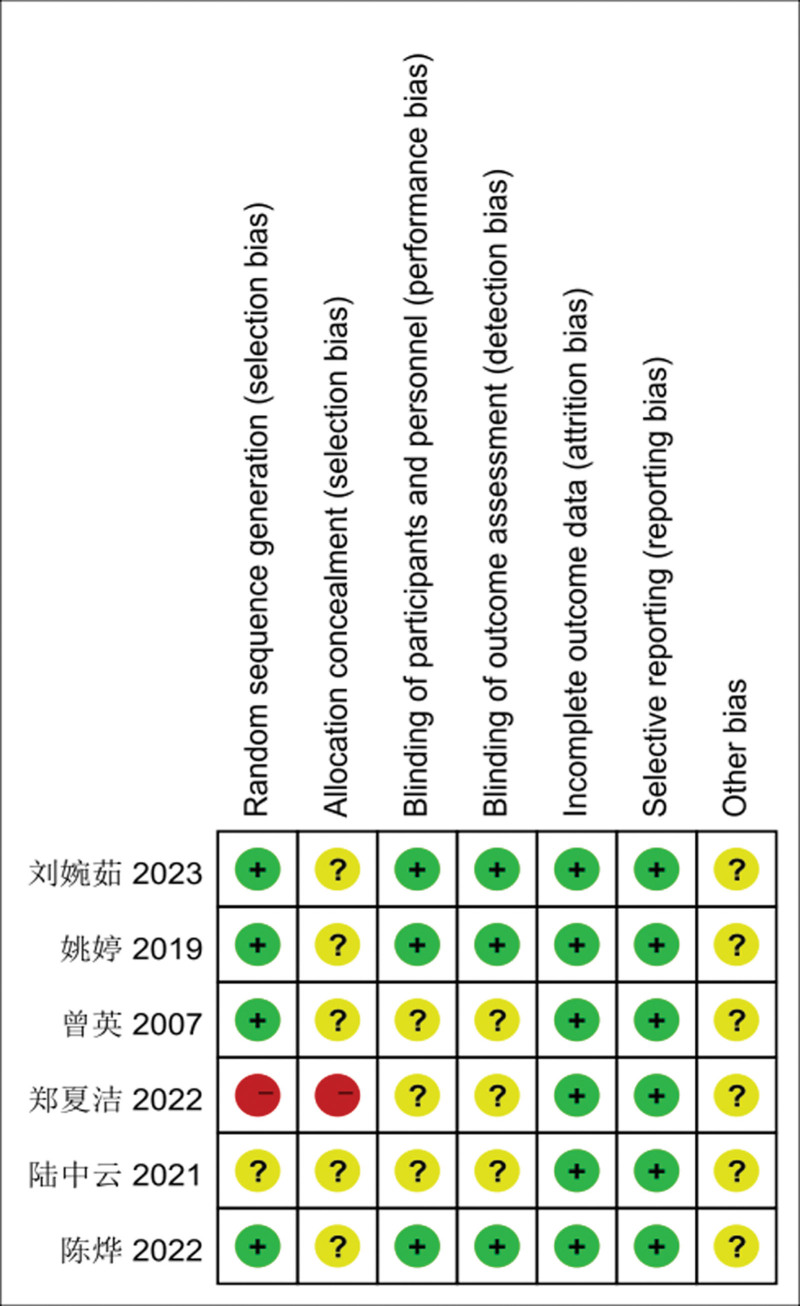
Risk of bias assessment for each study.

The quality of the 6 included literature^[3–8]^ was evaluated and the results of the quality evaluation are shown in Figure 2.

### 3.3. Meta-analysis results

#### 3.3.1. Comparison of serum TC levels before and after treatment

**Figure 3.**
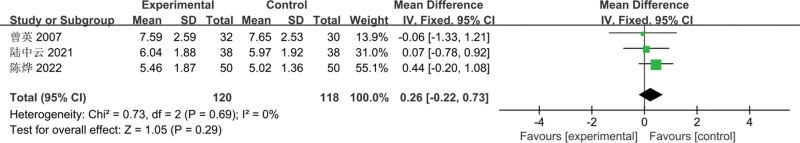
Meta-analysis of the comparison of serum TC levels before treatment between the 2 groups of patients. TC = total cholesterol.

**Figure 4.**
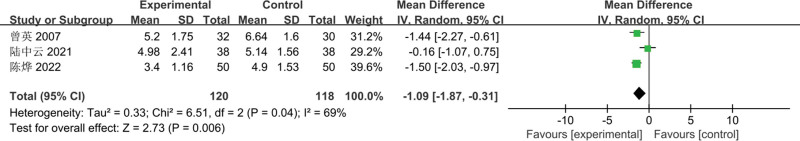
Meta-analysis of the comparison of serum TC levels between the 2 groups of patients after treatment. TC = total cholesterol.

The 3 RCTs^[4,7,8]^ included involved a total of 238 patients (120 cases in the experimental group and 118 cases in the control group); meta-analysis of the comparison of serum TC levels before treatment, using a fixed-effects model, there was no heterogeneity between studies (*I*^2^ = 0%, *P* = .69); the difference in TC levels between the 2 groups before treatment was not statistically significant [MD = 0.26, 95% CI: (−0.22 to 0.73, *P* = .29)], as shown in Figure 3. Meta-analysis of the comparison of TC levels between the 2 treatment groups, using a random-effects model, showed moderate heterogeneity among studies (*I*^2^ = 69%, *P* = .04); the difference in TC levels between the 2 treatment groups was statistically significant [MD = −1.09, 95% CI: (−1.87 to −0.31, *P* = .006)], as shown in Figure 4.

#### 3.3.2. Comparison of serum TG levels before and after treatment

**Figure 5.**
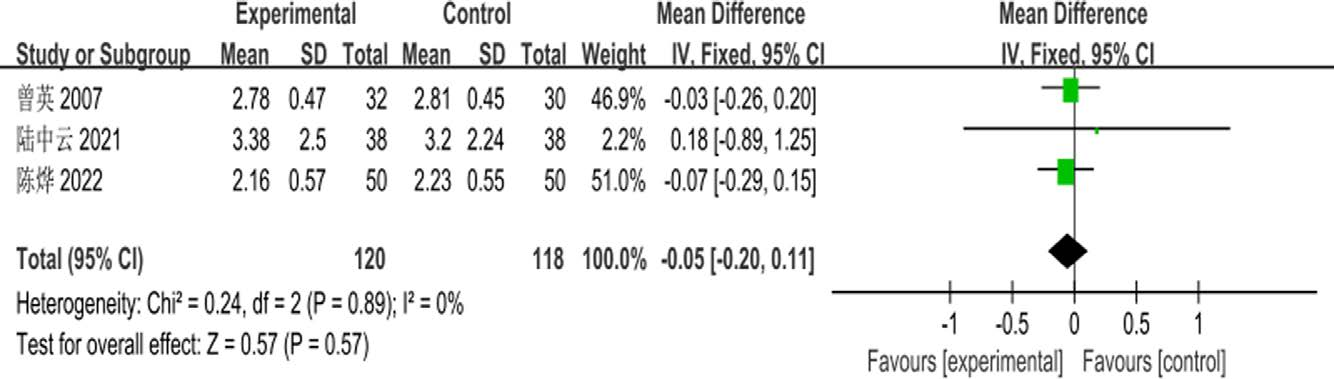
Meta-analysis of the comparison of serum TG levels before treatment between the 2 groups of patients. TG = triglyceride.

**Figure 6.**
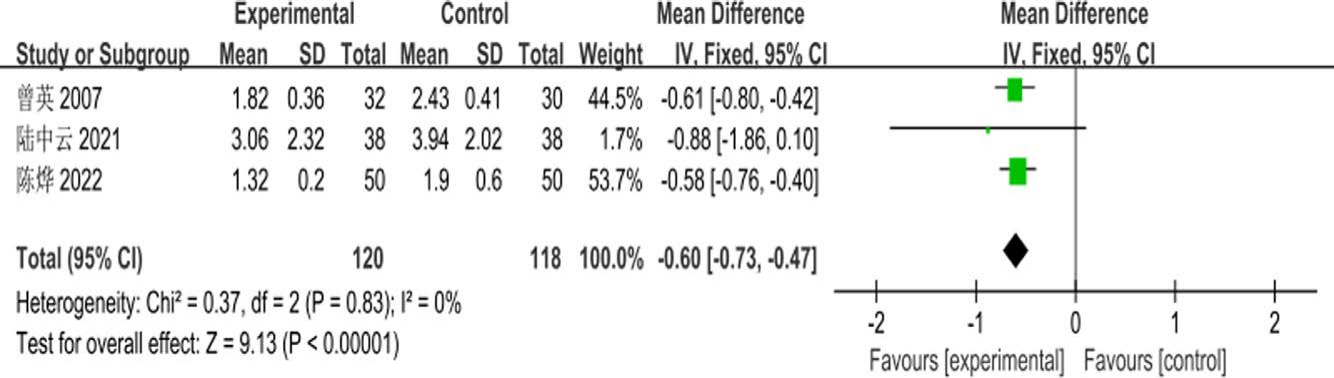
Meta-analysis of the comparison of serum TG levels after treatment in the 2 groups of patients. TG = triglyceride.

The 3 RCTs^[4,7,8]^ included involved a total of 238 patients (120 cases in the experimental group and 118 cases in the control group); meta-analysis for the comparison of serum TG levels before treatment was performed with a fixed-effects model, and there was no heterogeneity between studies (*I*^2^ = 0%, *P* = .89); the difference in TG levels between the 2 groups before treatment was not statistically significant [MD = −0.05, 95% CI: (−0.20 to 0.11, *P* = .57)], as shown in Figure 5. Meta-analysis of the comparison of posttreatment TG levels between the 2 groups, using a fixed-effects model, with no heterogeneity among studies (*I*^2^ = 0%, *P* = .83); the difference in posttreatment TG levels between the 2 groups was statistically significant [MD = −0.60, 95% CI: (−0.73 to −0.47, *P* < .00001)], as shown in Figure 6.

#### 3.3.3. Comparison of serum LDL-C levels before and after treatment

**Figure 7.**
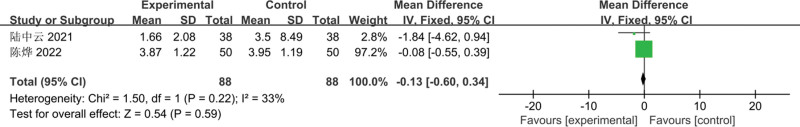
Meta-analysis of the comparison of pretreatment serum LDL-C levels between the 2 groups of patients. LDL-C = low-density lipoprotein cholesterol.

**Figure 8.**
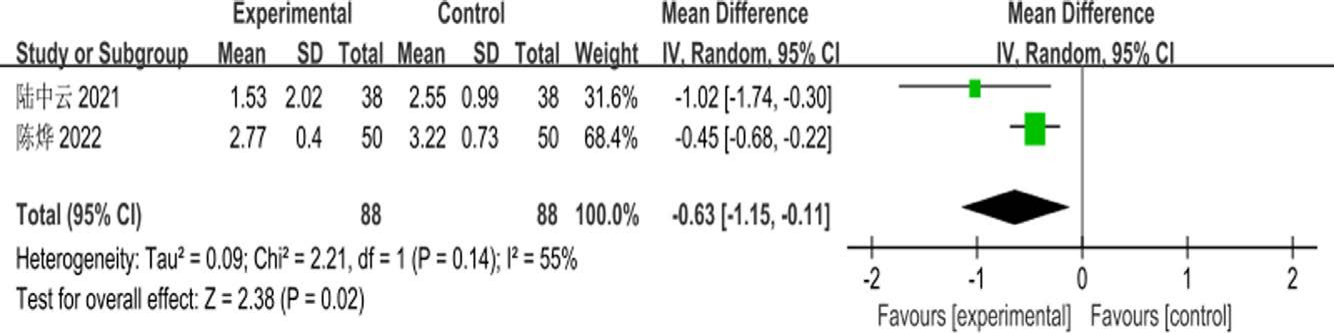
Meta-analysis of the comparison of serum LDL-C levels between the 2 groups of patients after treatment. LDL-C = low-density lipoprotein cholesterol.

The 2 included RCTs^[7,8]^ involved a total of 176 patients (88 patients in the experimental group and 88 patients in the control group); meta-analysis of the comparison of serum LDL-C levels before treatment was performed with a fixed-effects model, and there was a mild degree of heterogeneity between studies (*I*^2^ = 33%, *P* = .22); the difference in pretreatment LDL-C levels between the 2 groups had no statistical significance [MD = −0.13, 95% CI: (−0.60 to 0.34, *P* = .59)] as shown in Figure 7. Meta-analysis of the comparison of posttreatment LDL-C levels between the 2 groups, using a random-effects model, showed moderate heterogeneity among studies (*I*^2^ = 55%, *P* = .14); the difference in posttreatment LDL-C levels between the 2 groups was statistically significant [MD = −0.63, 95% CI: (−1.15 to −0.11, *P* = .02)], as shown in Figure 8.

#### 3.3.4. Comparison of BMI before and after treatment

**Figure 9.**
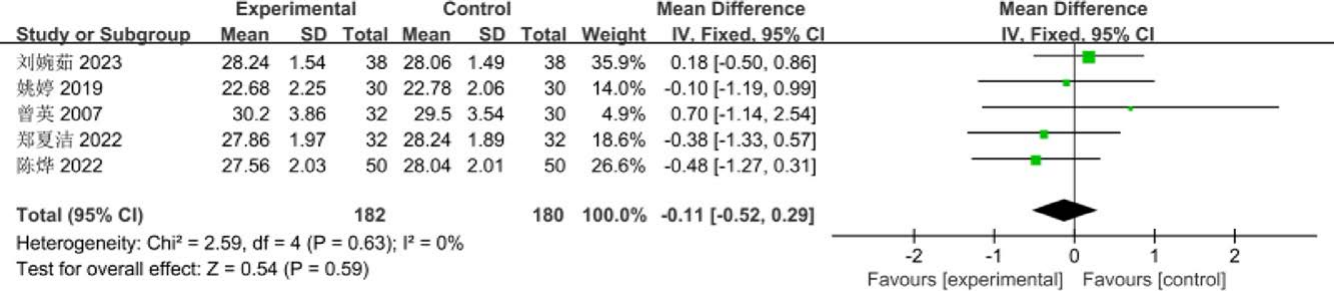
Meta-analysis of the comparison of pretreatment BMI between the 2 groups of patients. BMI = body mass index.

**Figure 10.**
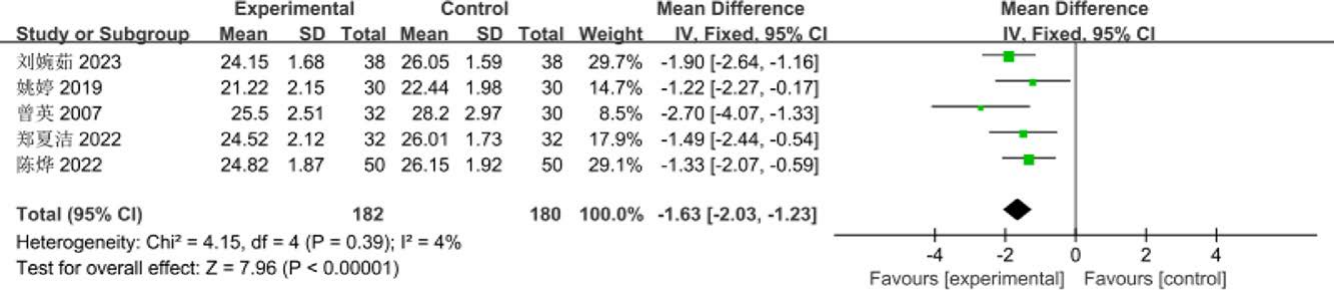
Meta-analysis of the comparison of posttreatment BMI between the 2 groups of patients. BMI = body mass index.

The 5 RCTs^[3–7]^ included involved a total of 362 patients (182 cases in the experimental group and 180 cases in the control group); Meta-analysis of the comparison of BMI before treatment, using a fixed-effects model, there was no heterogeneity between the studies (*I*^2^ = 0%, *P* = .63); the difference between the 2 groups of pretreatment BMI was not statistically significant [MD = −0.11, 95% CI: (−0.52 to 0.29, *P* = .59)], as shown in Figure 9. Meta-analysis of posttreatment BMI comparisons, using a fixed-effects model, showed no heterogeneity between studies (*I*^2^ = 4%, *P* = .39); the difference in posttreatment BMI between the 2 groups was not statistically significant [MD = −1.63, 95% CI: (−2.03 to −1.23, *P* < .00001)], as shown in Figure 10.

#### 3.3.5. Comparison of VFA before and after treatment

**Figure 11.**
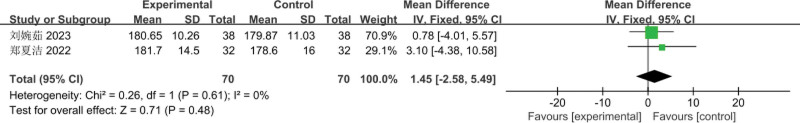
Meta-analysis comparing pretreatment VFA in the 2 groups of patients. VFA = visceral fat area.

**Figure 12.**
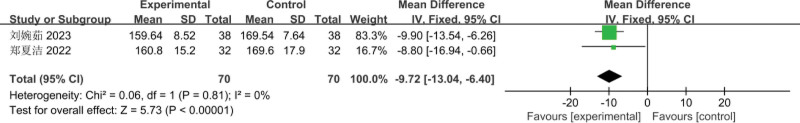
Meta-analysis comparing VFA after treatment in the 2 groups of patients. VFA = visceral fat area.

The 2 RCTs^[5,6]^ included involved a total of 140 patients (70 patients in the experimental group and 70 patients in the control group); meta-analysis of the comparison of serum VFA levels before treatment, using a fixed-effects model, there was no heterogeneity between studies (*I*^2^ = 0%, *P* = .61); the difference in the pretreatment LDL-C levels between the 2 groups was not statistically significant [MD = 1.45, 95% CI: (−2.58 to 5.49, *P* = .48)] as shown in Figure 11. Meta-analysis of the comparison of serum VFA levels between the 2 groups after treatment, using a fixed-effects model, showed no heterogeneity between studies (*I*^2^ = 0%, *P* = .81); the difference in the pretreatment LDL-C levels of the patients in the 2 groups, was not statistically significant [MD = −9.72, 95% CI: (−13.04 to −6.40, *P* < .00001)], as shown in Figure 12.

### 3.4. Publication bias analysis based on BMI, VFA outcomes

The distribution of the included studies on both sides of the funnel was largely symmetrical, suggesting that there was a low likelihood of publication bias. As in Figures 1 and 14.

**Figure 13.**
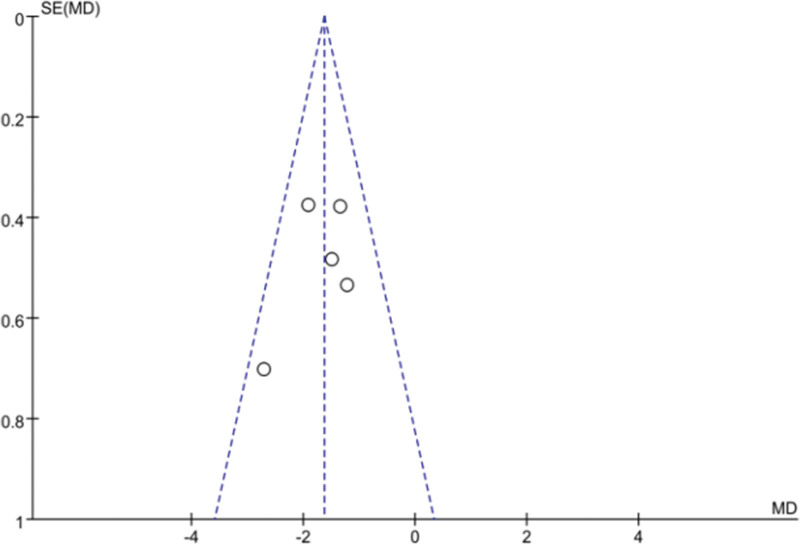
Funnel plot of publication bias test based on BMI outcome. BMI = body mass index.

**Figure 14.**
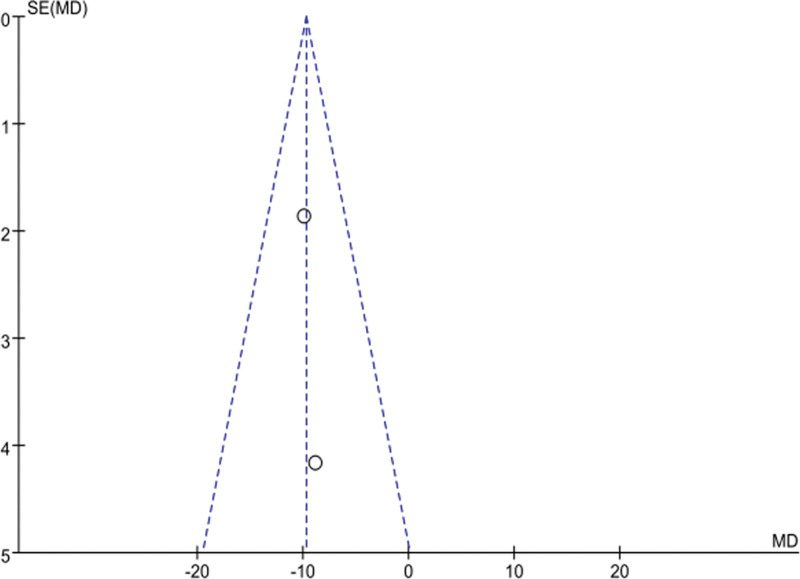
Funnel plot of publication bias test based on VFA outcomes. VFA = visceral fat area.

## 4. Summary and outlook

Obesity is a metabolic disease caused by imbalance between energy intake and consumption.^[9,10]^ According to Chinese medicine, obesity is mainly caused by the spleen, stomach and skin, and phlegm-dampness is the key to its pathology, and the development trend of obesity is “phlegm stasis, phlegm stasis and qi stagnation.”^[11]^ Complications of obesity pose a threat to health,^[12]^ such as hypertension, diabetes, etc, are closely related to obesity,^[13,14]^ sometimes affecting respiratory function.^[15]^ Weight control through TCM therapy is a robust program with long-term value. For a long time, Chinese medicine practitioners have developed a large number of Chinese medicine prescriptions^[16,17]^ in the course of treating obesity, which are highly targeted. The Yellow Emperor’s Classic of Internal Medicine says, “Flesh is firm, skin is full of fat; flesh is not firm, skin is slow to paste; skin and flesh are not separated from the meat. Cream more gas and skin long slow, so can longitudinal abdominal hanging fat; meat body capacity; fat its body to close the small”; now that “fat people” belongs to the overweight, not the full meaning of obesity; “meat people” belong to the tall and strong muscle People, body fat rate is not high; “cream people” often abdominal accumulation of large amounts of fat, belonging to the real obesity.^[18]^ Chinese medicine to explore its causative factors include: innate endowment, lack of exercise, dietary disorders, emotional and emotional discomfort, spleen and stomach weakness. Simple obesity is characterized by spleen deficiency and dampness obstruction, gastrointestinal heat, phlegm, stasis, etc. The main treatments include strengthening the spleen and dispelling dampness, clearing heat and removing toxins, and eliminating phlegm and stasis.^[19]^ In recent years, some hospitals in China have started to introduce weight loss tonics to achieve more lasting effects by conditioning the patient’s constitution and improving the patient’s own way, and it has the advantages of simple operation and small side effects. Yu et al^[20]^ believe that we should start from the perspective of strengthening the spleen, dispelling dampness and resolving phlegm, and through the regulation of cellular autophagy, thereby effectively improving obesity-related metabolic inflammatory diseases such as cardiovascular disease and diabetes. Li et al^[2]^ analyzed the causes of obesity from the perspectives of phlegm, deficiency, and depression, and proposed that traditional Chinese medicine soup can act on intestinal flora to treat obesity. Er Chen Tang is a traditional Chinese formula composed of Poria, Glycyrrhiza glabra, Semixia, and Pericarpium Citri Reticulatae, of which Pericarpium Citri Reticulatae and Semixia are better with age, hence the name Er Chen Tang. Its efficacy is mainly for drying dampness and resolving phlegm, regulating qi and harmonizing the middle. For simple obese patients, the significance is relatively limited, but for obese patients with dampness-heat internalization, their obesity is caused by excessive dampness in the body, and then Er Chen Tang can help patients expel dampness in the body and play the effect of weight loss, and the effect is good. This study analyzes the therapeutic efficacy of Er Chen Tang in the treatment of obesity, in order to add bricks and mortar to the clinical treatment of obesity in the future, to provide a more reliable evidence-based medicine, and to contribute to the weight loss of the whole population and public health.

A total of 6 RCTs with 438 obese patients were included in this meta-analysis, and the results of meta-analysis showed that the improvements in the levels of TC, TG, LDL-C, VFA, and BMI indexes of the experimental group were significantly better than those of the control group after treatment. It indicates that Er Chen Tang adjuvant treatment for obesity can promote recovery faster and possess better overall efficacy. In this meta-analysis, Er Chen Tang was supplemented with the traditional Chinese medicine soup Er Chen Tang on the basis of the original treatment, which was an enhancement and improvement of the original treatment, not a replacement of the original treatment.

There was moderate heterogeneity in the 2 indicators in this study, and after analysis, the reasons for this may be: there were differences in the age and gender composition of the patients included in each RCT; there were some differences in the physical conditions of the patients in each RCT; and there was a combination of medications and differences in the therapeutic efficacy of the treatments in some of the RCTs.

There are some limitations in this meta-analysis: the sample size of RCTs is small, which may have some impact on the evaluation of results; only 2 of the 6 RCTs mentioned the effectiveness rate, but the reference indexes were different, so the effectiveness rate was not dichotomized and discussed; only 4 of them reported the randomized method, and 3 RCTs did not explicitly state that they used blinding, so they may be biased in the study; and the conclusions may be controversial due to the lack of foreign RCTs of RCTs of Er Chen Tang for the treatment of obesity, the conclusion of meta-analysis may be controversial.

In conclusion, Er Chen Tang can improve obesity symptoms faster and has better efficacy, which is worth applying and promoting, but it is necessary to design randomized controlled studies with larger samples to provide more high-quality research bases for the secondary evaluation, so as to better demonstrate the efficacy of Er Chen Tang on obesity and evaluate its promotion value.

## Author contributions

**Conceptualization:** Xueyan Lv.

**Data curation:** Xingyu Kang.
